# Supplementary material for: Differential regulation of OCT4 targets facilitates reacquisition of pluripotency
Source: Nat Commun. 2019 Sep 30;10:4444. doi: 10.1038/s41467-019-11741-5 (PMC6768871; doi:10.1038/s41467-019-11741-5)
Supplement: Supplementary file 1 — Supplementary Information [file 41467_2019_11741_MOESM1_ESM.pdf]

Supplementary Information

Differential regulation of *OCT4* targets facilitates reacquisition of pluripotency

Thakurela *et al.*

List of Content

Supplementary Figures 1-5

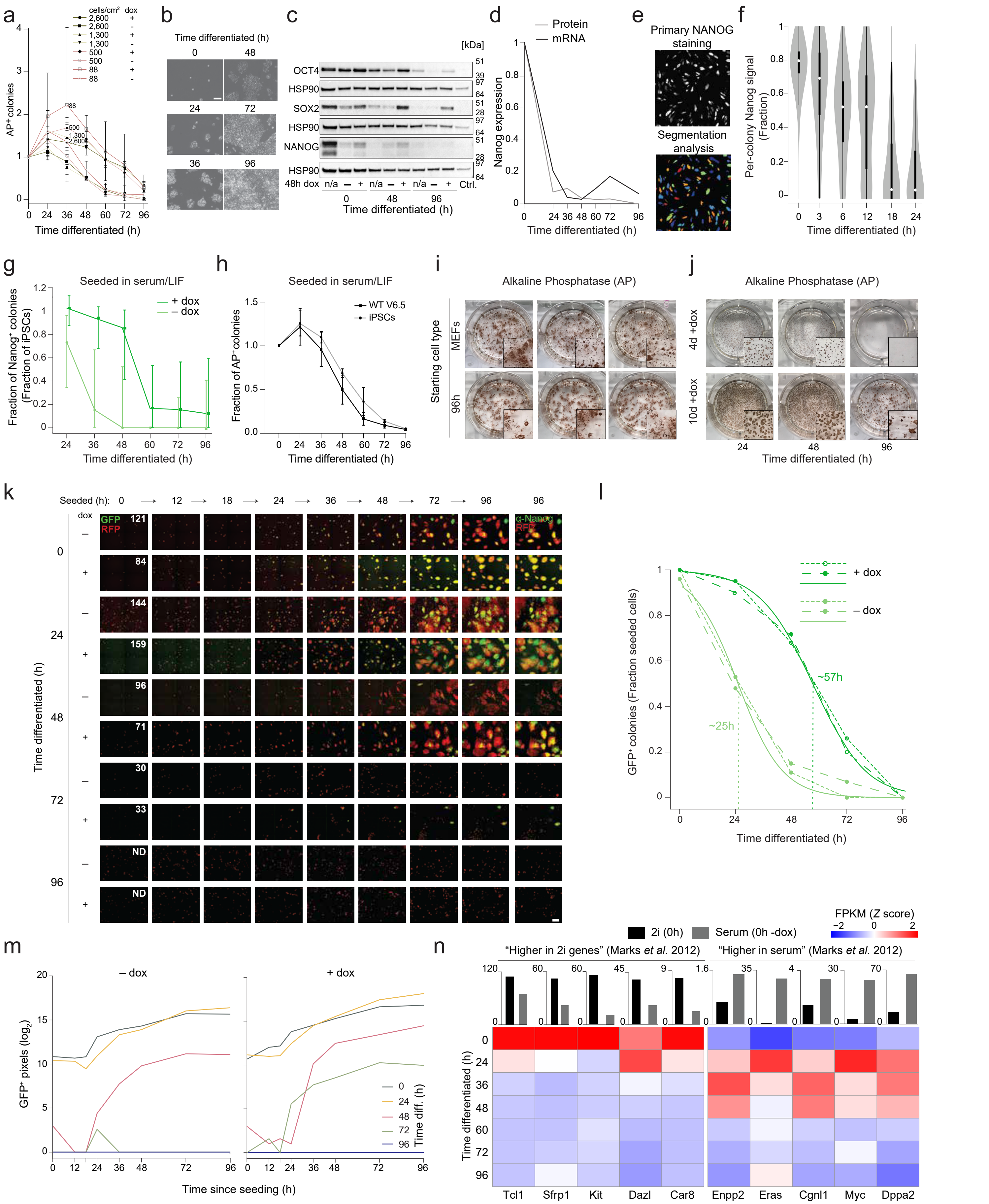

Supplementary Figure. 1: Characterizing the transition between high and low efficiency reprogramming

a. Plot showing normalized alkaline phosphatase (AP) positive colony count for cells diferentiated to selected time points and re-seeded at indicated densities.
b. Representative phase-contrast images (10x) of secondary iPSCs at select time points following withdrawal of 2i/LIF conditions (scale bar = 100 μm).
c. Western blots during differentiation and reprogramming. Ctr: mouse embryonic fibroblasts (MEFs) from CD1 E13.5 embryos.
d. The percent of NANOG+ cells (FACS) and population-level transcription (RNA-seq) normalized to iPSCs (0h).
e. Representative CellProfiler output (bottom)[1] used to count NANOG+ colonies after 48h of differentiation from IF images (top). Colonies counted by the software are indicated by the colored patches in the bottom image.

f. Distribution of NANOG signal by immunostaining, in cells differentiated by 2i/LIF withdrawal. Signal intensity is normalized to the median intensity for undifferentiated iPSC colonies in 2i/LIF media and represented as a fraction with respect to undifferentiated iPSCs.
g. Efficiency of colony forming ability generated from IF images as shown in Figure 1b and normalized to iPSC controls. Plot shows mean efficiencies for three biological replicates, and error bars represent standard deviation over four technical replicates per experiment per time point. Error bars for + dox conditions are shifted to facilitate visualization. Note that error bars are shown as normalized ratio of the specific time-point with respect to 0h, hence in some cases error bars may extend beyond 1.
h. Normalized values for alkaline phosphatase (AP) positive colonies for our (Nanog heterozygous) and V6.5 cell line (Nanog wild-type).
i. Alkaline phosphatase (AP) staining of iPSC colonies formed after 12 days of dox-induced reprogramming from either secondary inducible MEFs generated from our iPSC line (top row) or of iPSCs differentiated for 96h (bottom row) prior to dox induction. After 96h of differentiation and an additional 96h of dox, only 0.6% (+/- 0.53%) of dox-induced cells yields NANOG+ colonies, with minimal additional colonies formed over an extended 12-day reprogramming timeline (also see Figure 1e).
j. Alkaline phosphatase (AP) staining of iPSC colonies of cells differentiated for 24, 48, or 96h and reseeded in serum/LIF conditions with dox (4 days or 10 days).
k. Representative fields of lineages (defined as the colony formed from a re-seeded single cell) stemming from cells differentiated for 0, 24, 48, 72, or 96h, seeded at clonal density into – dox or + dox conditions, and tracked for 96h. Images shown are overlays between constitutive RFP and Nanog::GFP reporters. Right-most column is NANOG protein immunofluorescence and constitutive RFP at the end of the tracking period. Numbers indicate total lineages tracked, where the 48hr time point was used to define lineage identity. ND for the 96hr series denotes all colonies formed were below the threshold size.
Scale bar = 200 μm.
l. Percentage of lineages that generate NANOG+ colonies within 96h of seeding calculated from two independent experiments for – dox (light green) and + dox (dark green) conditions. Solid lines represent respective fitted sigmoid curves, with estimated midpoints (25h and 57h) that are nearly identical to those made from static counts (Figure 1d and Supplementary Figure 1g).
m. GFP+ (signal > 2,000 AU) pixels within the full imaged field from the Nanog::GFP reporter as a function of time post re-seeding for cells differentiated and re-seeded as in Supplementary Fig. 1l into – dox (left) and + dox (right) conditions.
n. Heatmaps show expression of select genes over our differentiation time course. Left: pluripotent state genes lost over differentiation (see Figure 3a).
Right: transiently upregulated genes. Bar plots show expression of same genes as shown heatmap below for pluripotent cells in LIF/2i conditions (0h time point) and serum/LIF media (0h + 48h no dox time point).

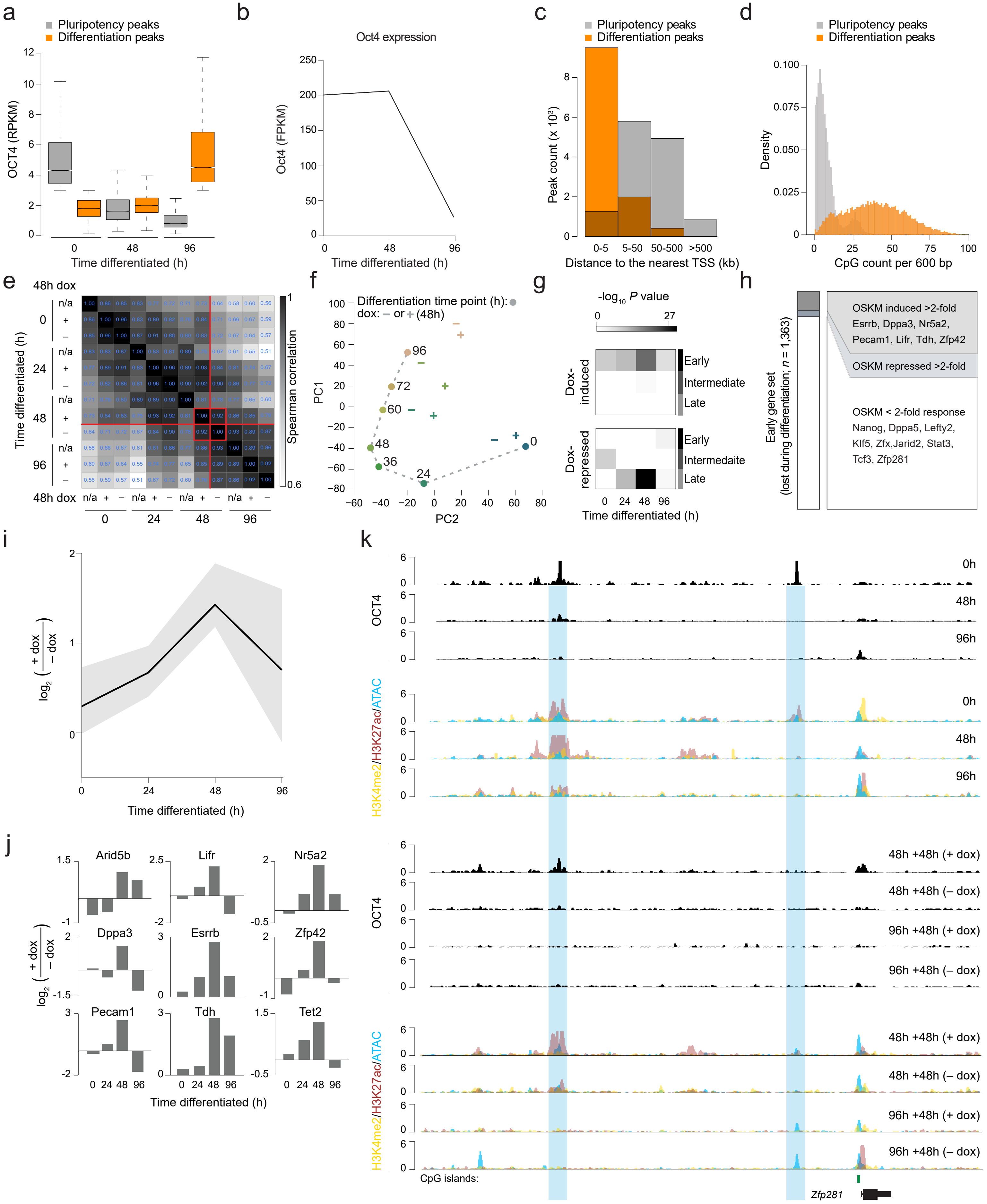

Supplementary Figure. 2: Gene expression and OCT4 binding dynamics

a. Boxplots of peak intensity (RPKM) for pluripotent (grey) and differentiation (orange) associated OCT4 peaks. Horizontal black lines in boxplots indicate medians; boxes represent interquartile range showing central 50% of data and whiskers indicate 25th and 75th percentile data.

b. Oct4 gene expression levels (RNA-seq) over undirected differentiation (FPKM = 25.4 at 96h). FPKM: Fragments per kilobase per million.

c. Histogram of the distance to the nearest annotated transcription start site (TSS) for pluripotency (grey) and differentiation associated (orange) OCT4 peak sets.

d. Histogram of CpG density for the pluripotent peaks (grey) and differentiation-associated (orange) OCT4 peak sets.

e. Sample correlation (Spearman) for differentiation ("n/a") and reprogramming ("+") RNA-seq time course, including – dox controls ("–"). Red boxes delineate samples that exhibit higher inter-sample Spearman correlations. Text in each box shows Spearman correlation.
f. Principal component analysis of dynamically expressed genes in differentiating cells (circles), cells induced to reprogram for 48h (dox; represented as "+"), and matched –dox controls (represented as "–"), shown as a trajectory in 2-dimensional space. Color-coding corresponds to the differentiation time point.
g.–log10 *P* value heatmap of the statistical overlap between genes that are dynamically expressed over differentiation and those that are ≥2-fold up- (top) or down- (bottom) regulated in response to OSKM induction compared to time matched – dox controls. *P* value for overlap is calculated by the hypergeometric test.
h. Pluripotency-associated genes ("Early" group in Figure 3a) are subdivided according to their OSKM-specific transcriptional response following 48h of differentiation: 119 genes are induced ≥ 2-fold compared to the – dox control (light grey), 38 are repressed ≥ 2-fold (slate grey), and 1,206 do not meet either criterion (white). Selected genes are listed for each category.
i. Expression ratio between + dox and – dox conditions for genes that are dox-inducible after 48h of differentiation show that these genes maximally differ between conditions at this time point. The black line indicates median value while the boundaries of the shaded region show the 25th and 75th percentile.
j. Gene expression ratio between matched + dox or – dox samples for genes that are induced upon OSKM after 48h differentiation show that these genes are selectively upregulated by ectopic OSKM specifically at this time point.
k. Representative browser tracks of exclusive and reaccessed peaks (as defined in Figure 4c) located at the Zfp281: OCT4, black; H3K4me2, yellow; H3K27ac, dark red; ATAC, blue). CpG island track is shown below the browser track with one CpG island (green box) near the Zfp281 TSS.

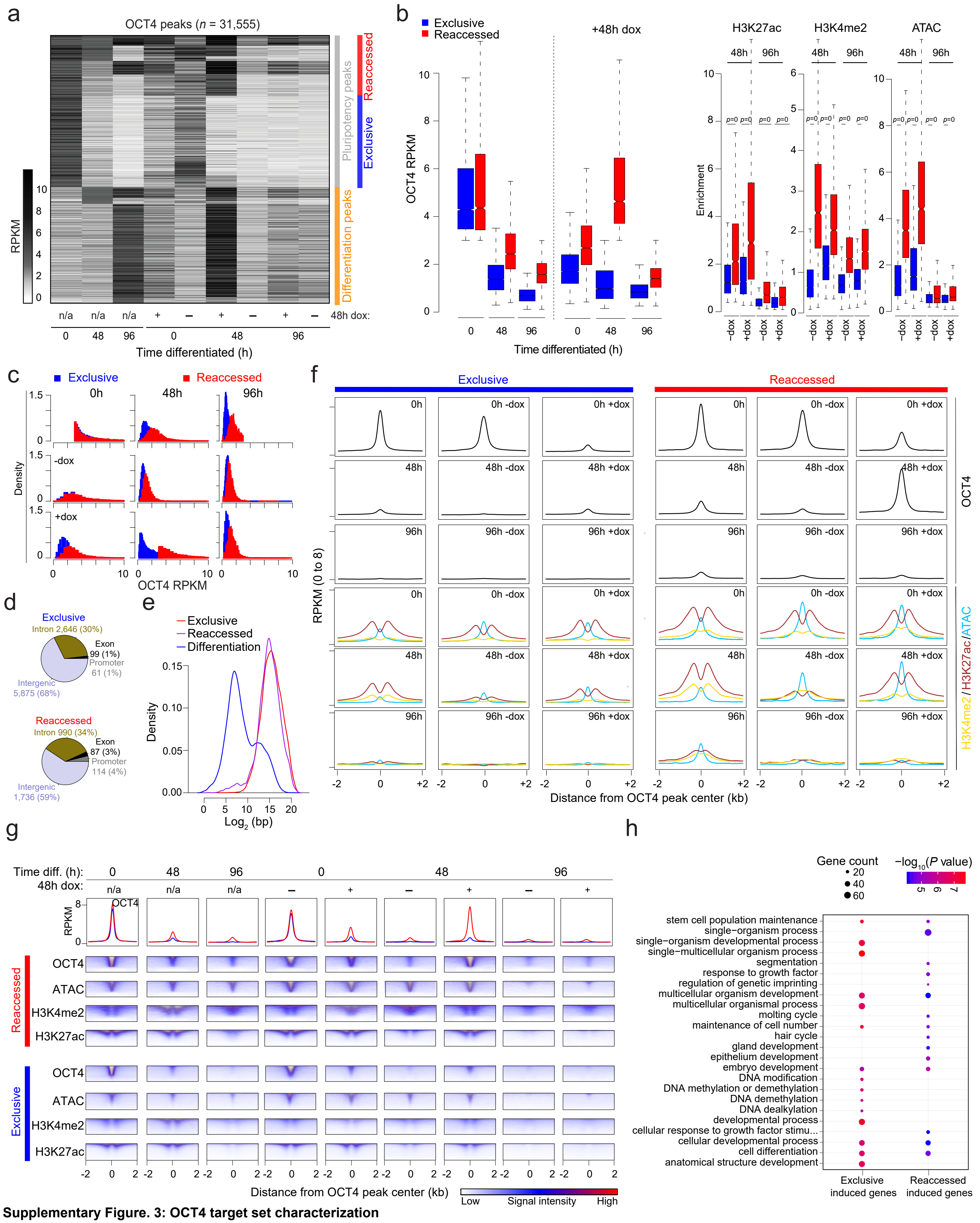

Supplementary Figure. 3: OCT4 target set characterization

a. OCT4 occupancy for all identified peaks over differentiation and reprogramming time points as determined by ChIP-seq. Peak intensity is shown as normalized RPKM value, with the strongest heat (black) capped at the 99th percentile of RPKM. Dynamics are labeled according to OCT4 enrichment during differentiation and reprogramming time-course. Pluripotency-associated peaks (as defined in Figure 4a) are subdivided into two categories based on their response to OSKM induction. A specific set of pluripotent peaks are reaccessed in the 48h + dox condition and designated as "reaccessed" (red), while the other pluripotent set remain exclusive to the pluripotent state and designated as "exclusive" (blue). RPKM: Reads per kilobase per million.

b. Boxplots of OCT4 enrichment at exclusive (blue) and reaccessed (red) peaks. Left panel: Boxplots of OCT4 enrichment (RPKM) during differentiation and in the 48h “+ dox”. Box width is directly proportional to the number of peaks in each set. Right panel: Boxplots showing enrichment for H3K27ac, H3K4me2 and ATA-seq signal in “– dox” and “+ dox” conditions. Horizontal black lines in boxplots indicate medians; boxes represent interquartile range showing central 50% of data and whiskers indicate 25th and 75th percentile data.
c. Density histograms showing the distribution of OCT4 peak enrichments (RPKM) for differentiation (top), – dox (middle) and + dox (bottom) samples at exclusive (blue) and reaccessed (red) sites. X-axis is capped at 10 to remove outliers.
d. Pie charts showing the genomic distribution of exclusive and reaccessed peaks.
e. Density plot showing distance to the nearest TSS (x-axis, log(2)) for exclusive, reaccessed, and differentiation associated peaks.
f. Composite plots of OCT4 enrichment and chromatin dynamics (H3K4me2 and H3K27ac as well as chromatin accessibility, as measured by ATAC-seq) within +/– 2 kb of the peak center for exclusive (left panel) and reaccessed (right panel) OCT4 targets. Color code: OCT4 (black), H3K4me2 (yellow), H3K27ac (dark red), and ATAC (blue).
g. Top: composite plots of the OCT4 enrichment (RPKM) at exclusive (blue) and reaccessed (red) peaks for all differentiation (n/a), – dox (–), and + dox (+) samples. Below: read density heatmaps for OCT4, H3K4me2, H3K27ac, and ATAC-seq signal over the same peak sets. The signal intensity for each category is internally normalized and kept to the 99th percentile of the max read density observed across all samples from the union of exclusive and reaccessed OCT4 peak sets.
h. Plot showing Gene Ontology (GO) enrichment for biological pathways for the set of genes that are near to “exclusive” or “reaccessed” peaks and are induced in the 48h “+ dox” condition. Size of the dots represents number of genes in respective category and color indicates P value.

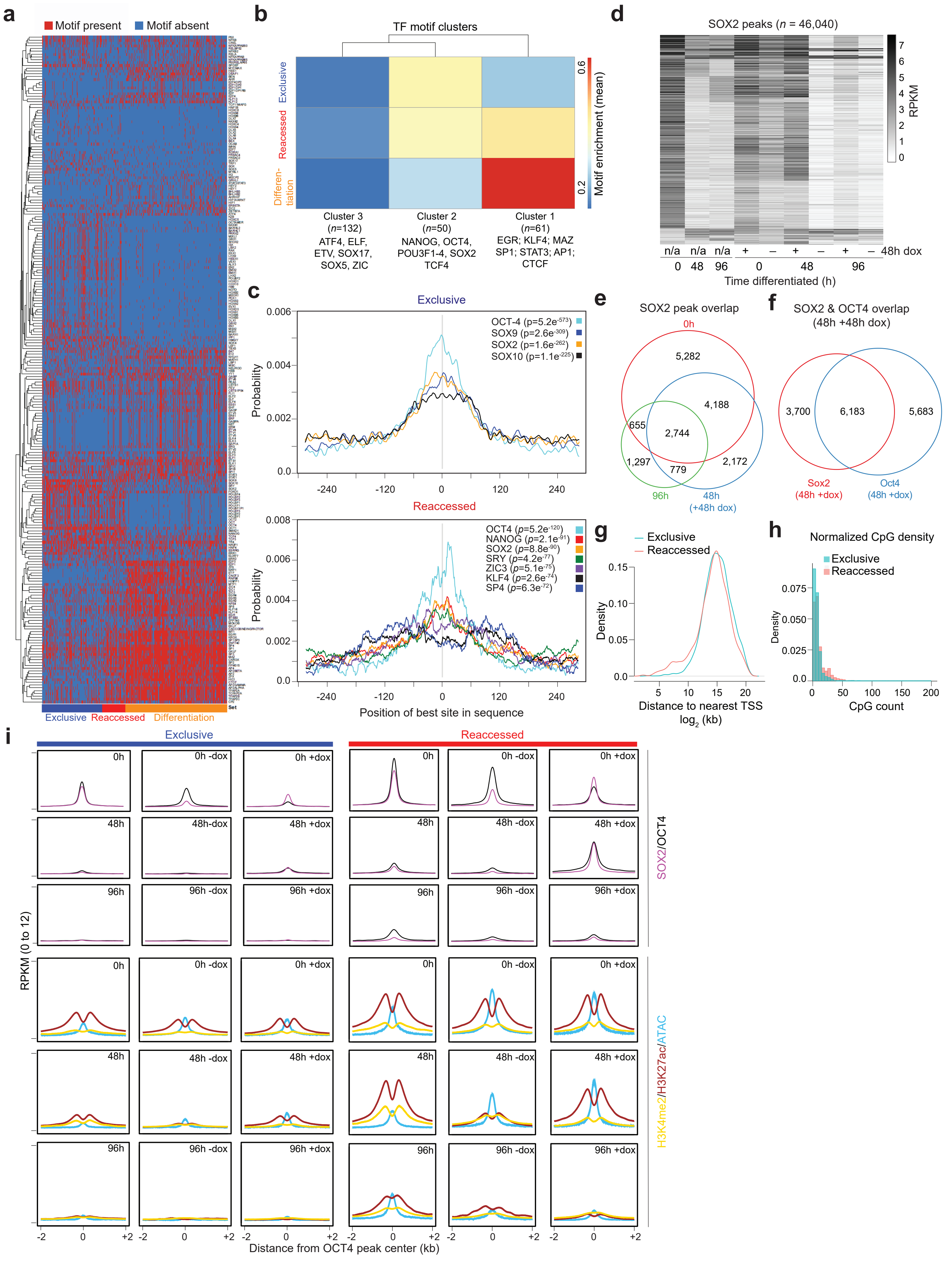
a
Motif present
Motif absent
Exclusive
Reaccessed
Differentiation
Set
b
TF motif clusters
Exclusive
Reaccessed
Differen-tiation
Cluster 3 (n=132) ATF4, ELF, ETV, SOX17, SOX5, ZIC
Cluster 2 (n=50) NANOG, OCT4, POU3F1-4, SOX2 TCF4
Cluster 1 (n=61) EGR; KLF4; MAZ SP1; STAT3; AP1; CTCF
Motif enrichment (mean)
0.6
0.2
c
Exclusive
OCT-4 (p=5.2e-573)
SOX9 (p=2.6e-309)
SOX2 (p=1.6e-262)
SOX10 (p=1.1e-225)
Probability
Reaccessed
OCT4 (p=5.2e-120)
NANOG (p=2.1e-91)
SOX2 (p=8.8e-90)
SRY (p=4.2e-77)
ZIC3 (p=5.1e-75)
KLF4 (p=2.6e-74)
SP4 (p=6.3e-72)
Position of best site in sequence
d
SOX2 peaks (n = 46,040)
RPKM
n/a n/a n/a + – + – + – 48h dox
0 48 96 0 48 96
Time differentiated (h)
e
SOX2 peak overlap
0h
5,282
4,188
655
2,744
1,297
779
2,172
96h
48h (+48h dox)
f
SOX2 & OCT4 overlap (48h +48h dox)
3,700
6,183
5,683
Sox2 (48h +dox)
Oct4 (48h +dox)
g
Exclusive
Reaccessed
Density
Distance to nearest TSS log2 (kb)
h
Normalized CpG density
Exclusive
Reaccessed
Density
CpG count
i
Exclusive
Reaccessed
0h
0h -dox
0h +dox
48h
48h-dox
48h -dox
48h +dox
96h
96h -dox
96h +dox
SOX2/OCT4
H3K4me2/H3K27ac/ATAC
RPKM (0 to 12)
Distance from OCT4 peak center (kb)

Supplementary Figure. 4: Co-factors might facilitate prolonged OCT4 dynamics at reaccessed sites

a. Heatmap showing binarized representation of the presence or absence of TF motifs for three peak sets. A hierarchical clustering was performed to cluster the TFs. Blue and red indicates the absence or presence of a TF motif.

b. The binary matrix used in panel "**a**" was clustered using k-means (k=3) to identify cluster of TFs enriched in the three peak sets. Representative motifs from each cluster are indicated below each cluster.

c. Plots showing location preferences of identified motifs in exclusive and reaccessed peaks using CentriMo[2].

d. Heatmap showing clustering of SOX2 peaks. Analysis for SOX2 was performed in same way as reported for OCT4 in Supplementary Figure 3a.

e. Venn diagram of SOX2 peaks in iPSCs (0h; blue) and the 96h differentiated cells (yellow) as well as for cells exposed to dox for 48h after 48h of differentiation (light green, 48h + 48h dox).

f. Venn diagram showing overlap of SOX2 and OCT4 peaks from cells differentiated for 48h and induced by OSKM to reacquire pluripotency.

g. Density plot showing distance (log2(kb)) to the nearest TSS for the exclusive and reaccessed set of SOX2 peaks.

h. Density plot showing CpG count for the exclusive and reaccessed set of SOX2 peaks.

i. Composite plots as shown in Supplementary Fig. 3f but for SOX2 peaks.

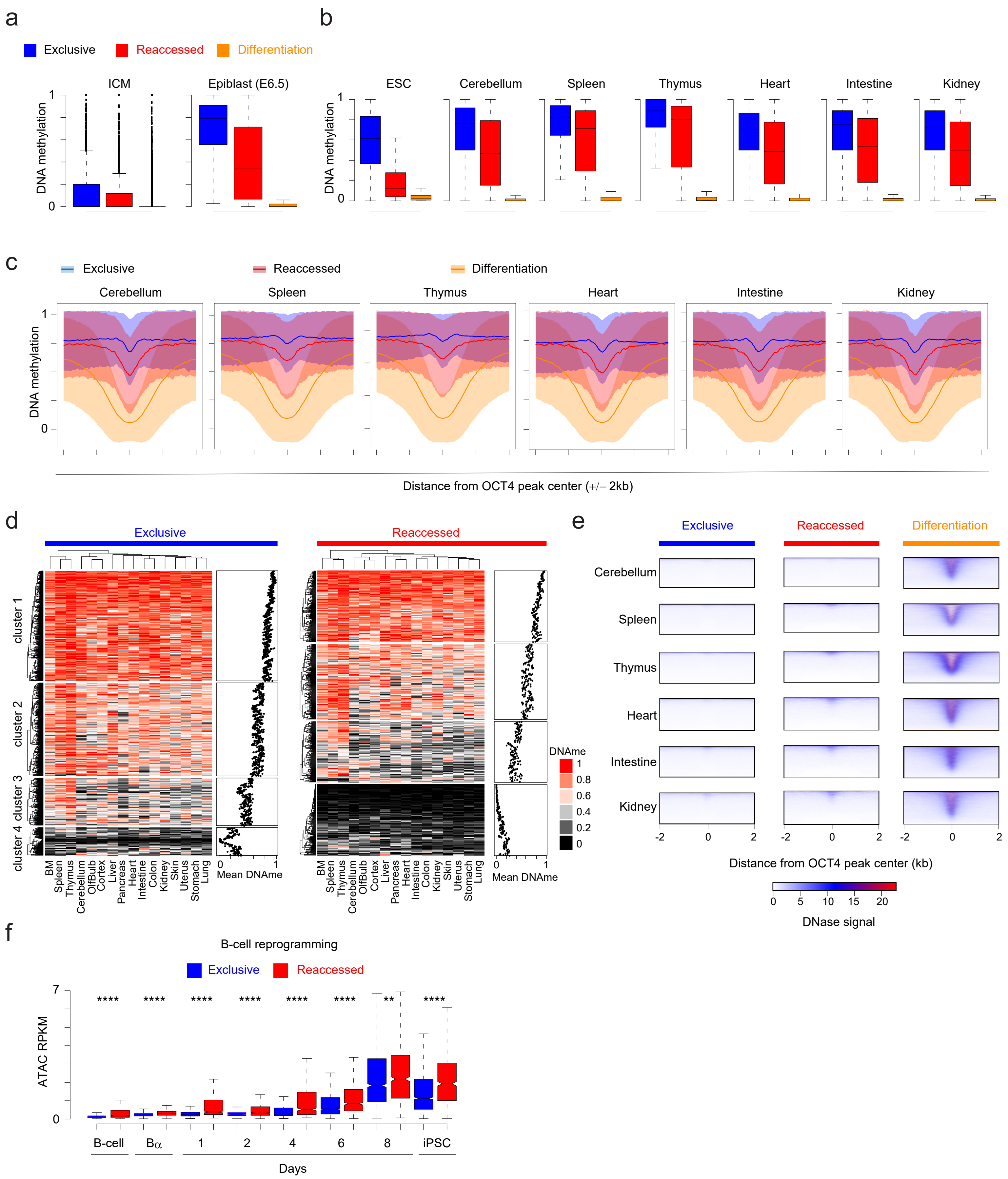

Supplementary Figure. 5: Somatic hypomethylation of select OCT4 targets

a. DNA methylation boxplots as measured by WGBS, shown for exclusive, reaccessed, and differentiation-associated OCT4 targets in ICM (E3.5) and epiblast (E6.5). Horizontal black lines in boxplots indicate medians; boxes represent interquartile range showing central 50% of data and whiskers indicate 25th and 75th percentile data.

b. Same as in Supplementary 5a but for mESCs and six selected somatic tissues. Data sets were obtained from the ENCODE consortium[3].

c. Composite plots for DNA methylation within +/– 2kb of each OCT4 peak set for the six somatic tissue types (shown as an averaged value in Figure 5b). The lines show median values while the shaded area represents +/– 1 standard deviation.

d. k-means clustering heatmap (k=4) of DNA methylation levels from an extended set of somatic tissues to identify regions that consistently show low methylation levels across somatic tissues. Dot plots to the right show mean methylation value across tissues for individual regions.

e. DNase-seq signal at the three OCT4 peak sets within the somatic tissues (shown as an averaged value in Figure 5b). OCT4 peaks were extended to +/– 2kb from the peak center and then mean values for DNase signal was calculated.
f. Boxplots showing ATAC-seq enrichments (RPKM) at the exclusive and reaccessed set of peaks from re-analysis of a recent B-cell reprogramming study[4]. Horizontal black lines in boxplots indicate medians; boxes represent interquartile range showing central 50% of data and whiskers indicate 25th and 75th percentile dat[illegible]

Supplementary References

1 Carpenter, A. E. et al. CellProfiler: image analysis software for identifying and quantifying cell phenotypes. Genome Biol 7, R100, doi:10.1186/gb-2006-7-10-r100 (2006).
2 Bailey, T. L. & Machanick, P. Inferring direct DNA binding from ChIP-seq. Nucleic Acids Res 40, e128, doi:10.1093/nar/gks433 (2012).
3 Yue, F. et al. A comparative encyclopedia of DNA elements in the mouse genome. Nature 515, 355-364, doi:10.1038/nature13992 (2014).
4 Stadhouders, R. et al. Transcription factors orchestrate dynamic interplay between genome topology and gene regulation during cell reprogramming. Nat Genet 50, 238-249, doi:10.1038/s41588-017-0030-7 (2018).
