# Supplementary material for: Differential regulation of OCT4 targets facilitates reacquisition of pluripotency
Source: Nat Commun. 2019 Sep 30;10:4444. doi: 10.1038/s41467-019-11741-5 (PMC6768871; doi:10.1038/s41467-019-11741-5)
Supplement: Supplementary file 3 — Reporting Summary [file 41467_2019_11741_MOESM3_ESM.pdf]

nature research

Corresponding author(s): Iftach Nachman, Alexander Meissner

Last updated by author(s): Apr 11, 2019

Reporting Summary

Nature Research wishes to improve the reproducibility of the work that we publish. This form provides structure for consistency and transparency in reporting. For further information on Nature Research policies, see Authors & Referees and the Editorial Policy Checklist.

Statistics

For all statistical analyses, confirm that the following items are present in the figure legend, table legend, main text, or Methods section.

n/a	Confirmed	
☐	☒	The exact sample size (n) for each experimental group/condition, given as a discrete number and unit of measurement
☐	☒	A statement on whether measurements were taken from distinct samples or whether the same sample was measured repeatedly
☐	☒	The statistical test(s) used AND whether they are one- or two-sided<br>*Only common tests should be described solely by name; describe more complex techniques in the Methods section.*
☒	☐	A description of all covariates tested
☒	☐	A description of any assumptions or corrections, such as tests of normality and adjustment for multiple comparisons
☒	☐	A full description of the statistical parameters including central tendency (e.g. means) or other basic estimates (e.g. regression coefficient) AND variation (e.g. standard deviation) or associated estimates of uncertainty (e.g. confidence intervals)
☒	☐	For null hypothesis testing, the test statistic (e.g. F, t, r) with confidence intervals, effect sizes, degrees of freedom and P value noted<br>*Give P values as exact values whenever suitable.*
☒	☐	For Bayesian analysis, information on the choice of priors and Markov chain Monte Carlo settings
☒	☐	For hierarchical and complex designs, identification of the appropriate level for tests and full reporting of outcomes
☒	☐	Estimates of effect sizes (e.g. Cohen's d, Pearson's r), indicating how they were calculated

Our web collection on statistics for biologists contains articles on many of the points above.

Software and code

Policy information about availability of computer code

Data collection	*Provide a description of all commercial, open source and custom code used to collect the data in this study, specifying the version used OR state that no software was used.*
Data analysis	R (v3.4.1); TopHat(v2.0.14); Cufflinks(v2.2.1); Bowtie(v2.2.5); MACS(v2.1.0); QuasR(v1.16); Homer(v2); FIMO; GeneOverlap(v1.12); regioneR(1.8.1), MetaMorph, CellProfiler

For manuscripts utilizing custom algorithms or software that are central to the research but not yet described in published literature, software must be made available to editors/reviewers. We strongly encourage code deposition in a community repository (e.g. GitHub). See the Nature Research guidelines for submitting code & software for further information.

Data

Policy information about availability of data

All manuscripts must include a data availability statement. This statement should provide the following information, where applicable:

- Accession codes, unique identifiers, or web links for publicly available datasets
- A list of figures that have associated raw data
- A description of any restrictions on data availability

All data generated as part of this study have been deposited in GEO under accession number GSE117205. Additional published data sets used in this study can be found under the following accession numbers: GSE84236: DNAme (ICM(3.5) and Epiblast (6.5)); GSE67520 & GSE101905 : OCT4 ChIP-seq; GSE93029: ATAC-seq MEF (reprogramming); GSE96611 : ATAC-seq (B-cell reprogramming); GSE106838 : DNAme (MEF reprogramming); GSE42836 : DNAme (Somatic tissues); GSE30206 : DNAme (mESC); GSE49847 : DNase-seq.

Field-specific reporting

Please select the one below that is the best fit for your research. If you are not sure, read the appropriate sections before making your selection.

☒ Life sciences ☐ Behavioural & social sciences ☐ Ecological, evolutionary & environmental sciences

For a reference copy of the document with all sections, see nature.com/documents/nr-reporting-summary-flat.pdf

Life sciences study design

All studies must disclose on these points even when the disclosure is negative.

Sample size	n/a
Data exclusions	n/a
Replication	All experiments were conducted with at least 2 biological replicates.
Randomization	n/a
Blinding	n/a

Reporting for specific materials, systems and methods

We require information from authors about some types of materials, experimental systems and methods used in many studies. Here, indicate whether each material, system or method listed is relevant to your study. If you are not sure if a list item applies to your research, read the appropriate section before selecting a response.

Materials & experimental systems

n/a	Involved in the study	
☐	☒	Antibodies
☐	☒	Eukaryotic cell lines
☒	☐	Palaeontology
☒	☐	Animals and other organisms
☒	☐	Human research participants
☒	☐	Clinical data

Methods

n/a	Involved in the study	
☐	☒	ChIP-seq
☒	☐	Flow cytometry
☒	☐	MRI-based neuroimaging

Antibodies

Antibodies used	OCT4 (Santa Cruz, sc-8628x), SOX2 (Santa Cruz, sc-17319), H3K4me2 (Millipore, 07-030), H3K27ac (Diagenode, C15410196).
Validation	n/a

Eukaryotic cell lines

Policy information about cell lines

Cell line source(s)	*State the source of each cell line used.*
Authentication	n/a
Mycoplasma contamination	n/a
Commonly misidentified lines (See ICLAC register)	n/a

ChIP-seq

Data deposition

☒ Confirm that both raw and final processed data have been deposited in a public database such as GEO.

☒ Confirm that you have deposited or provided access to graph files (e.g. BED files) for the called peaks.

Data access links<br>*May remain private before publication.*	https://www.ncbi.nlm.nih.gov/geo/query/acc.cgi?acc=GSE117205
Files in database submission	ATAC_0h_Rep1; ATAC_0h_Rep2; ATAC_0h_NoDox_Rep1; ATAC_0h_NoDox_Rep2; ATAC_0h_Dox_Rep1; ATAC_0h_Dox_Rep2; ATAC_48h_Rep1; ATAC_48h_Rep2; ATAC_48h_NoDox_Rep1; ATAC_48h_NoDox_Rep2; ATAC_48h_Dox_Rep1; ATAC_48h_Dox_Rep2; ATAC_96h_Rep1; ATAC_96h_Rep2; ATAC_96h_NoDox_Rep1; ATAC_96h_Dox_Rep1; ATAC_96h_Dox_Rep2; ATAC_96h_Dox_Rep3; OCT4_ChIP_0h_Rep1; OCT4_ChIP_0h_Rep2; OCT4_ChIP_0h_NoDox_Rep1; OCT4_ChIP_0h_NoDox_Rep2; OCT4_ChIP_0h_Dox_Rep1; OCT4_ChIP_0h_Dox_Rep2; OCT4_ChIP_48h_Rep1; OCT4_ChIP_48h_Rep2; OCT4_ChIP_48h_NoDox_Rep1; OCT4_ChIP_48h_NoDox_Rep2; OCT4_ChIP_48h_Dox_Rep1; OCT4_ChIP_48h_Dox_Rep2; OCT4_ChIP_96h_Rep1; OCT4_ChIP_96h_Rep2; OCT4_ChIP_96h_NoDox_Rep1; OCT4_ChIP_96h_NoDox_Rep2; OCT4_ChIP_96h_Dox_Rep1; OCT4_ChIP_96h_Dox_Rep2; K27ac_ChIP_0h_Rep1; K27ac_ChIP_0h_Rep2; K27ac_ChIP_0h_NoDox_Rep1; K27ac_ChIP_0h_NoDox_Rep2; K27ac_ChIP_0h_Dox_Rep1; K27ac_ChIP_0h_Dox_Rep2; K27ac_ChIP_48h_Rep1; K27ac_ChIP_48h_Rep2; K27ac_ChIP_48h_NoDox_Rep1; K27ac_ChIP_48h_NoDox_Rep2; K27ac_ChIP_48h_Dox_Rep1; K27ac_ChIP_48h_Dox_Rep2; K27ac_ChIP_96h_Rep1; K27ac_ChIP_96h_Rep2; K27ac_ChIP_96h_NoDox_Rep1; K27ac_ChIP_96h_NoDox_Rep2; K27ac_ChIP_96h_Dox_Rep1; K4me2_ChIP_0h_Rep1; K4me2_ChIP_0h_Rep2; K4me2_ChIP_0h_NoDox_Rep1; K4me2_ChIP_0h_NoDox_Rep2; K4me2_ChIP_0h_Dox_Rep1; K4me2_ChIP_0h_Dox_Rep2; K4me2_ChIP_48h_Rep1; K4me2_ChIP_48h_Rep2; K4me2_ChIP_48h_NoDox_Rep1; K4me2_ChIP_48h_NoDox_Rep2; K4me2_ChIP_48h_Dox_Rep1; K4me2_ChIP_48h_Dox_Rep2; K4me2_ChIP_96h_Rep1; K4me2_ChIP_96h_Rep2; K4me2_ChIP_96h_NoDox_Rep1; K4me2_ChIP_96h_NoDox_Rep2; K4me2_ChIP_96h_Dox_Rep1; K4me2_ChIP_96h_Dox_Rep2; RNA_0h_rep1; RNA_0h_rep2; RNA_24h_rep1; RNA_24h_rep2; RNA_36h_rep1; RNA_36h_rep2; RNA_48h_rep1; RNA_48h_rep2; RNA_60h_rep1; RNA_60h_rep2; RNA_72h_rep1; RNA_72h_rep2; RNA_96h_rep1; RNA_96h_rep2; RNA_0h_NoDox_rep1; RNA_0h_NoDox_rep2; RNA_0h_Dox_rep1; RNA_0h_Dox_rep2; RNA_24h_NoDox_rep1; RNA_24h_NoDox_rep2; RNA_24h_Dox_rep1; RNA_24h_Dox_rep2; RNA_48h_NoDox_rep1; RNA_48h_NoDox_rep2; RNA_48h_Dox_rep1; RNA_48h_Dox_rep2; RNA_96h_NoDox_rep1; RNA_96h_NoDox_rep2; RNA_96h_Dox_rep1; RNA_96h_Dox_rep2; WCE
Genome browser session<br>(e.g. UCSC)	n/a

Methodology

Replicates	2
Sequencing depth	File provided as a supplementary table
Antibodies	OCT4 (Santa Cruz, sc-8628x), SOX2 (Santa Cruz, sc-17319), H3K4me2 (Millipore, 07-030), H3K27ac (Diagenode, C15410196).
Peak calling parameters	OCT4 binding sites were identified using the MACS v2.1.0 peak caller with the flags: "--bdg --gsize mm", an FDR < 0.05 and using a common whole cell extract BAM as the background for all time points. Peaks were called against a set of merged whole cell extract (WCE) reads generated by randomly sampling 10M reads from six different WCE samples. In order to track the dynamics of individual peaks over time, we devised the following strategy to merge peaks from different time points into an epitope-wide "consensus peak set." Peak summits called by MACS were merged into a consensus region if they fell within 1 bp of each other. A new summit location was determined by taking the weighted average of all peak summits within the consensus region. Following the designation of the new summit, the peak region was defined by extending outwards by 300 bp on either side of the summit. Peak intensities were defined as the maximum number of reads within the 600 bp peak region, normalized by length and library size to get an RPKM value.
Data quality	FASTQC
Software	MACS v2.1.0
