# Supplementary material for: Differential regulation of OCT4 targets facilitates reacquisition of pluripotency
Source: Nat Commun. 2019 Sep 30;10:4444. doi: 10.1038/s41467-019-11741-5 (PMC6768871; doi:10.1038/s41467-019-11741-5)
Supplement: Supplementary file 4 — Description of Additional Supplementary Files [file 41467_2019_11741_MOESM4_ESM.pdf]

Description of Additional Supplementary Files

File Name: Supplementary Data 1
Description: Table for RPKM values for RNA-seq datasets.

File Name: Supplementary Data 2
Description: Table for alignment statistics for ChIP-seq datasets.

File Name: Supplementary Data 3
Description: Table for RPKM values for OCT4 ChIP-seq datasets and histone modifications.

File Name: Supplementary Data 4
Description: Table showing presence or absence of transcription factor motifs at identified OCT4 peaks.

File Name: Supplementary Data 5
Description: Table for RPKM values for SOX2 ChIP-seq peaks.

File Name: Supplementary Movie 1
Description: Live tracking of pluripotency reacquisition assay for cells differentiated for 0 hours, and reseeded under Serum/LIF +OSKM (0h + dox). The cells (constitutively expressing RFP) were imaged live at 0,12,24,36,48,72 and 96 hours, and then again after staining at 96 hrs for Nanog (green). For the purpose of statistics (Fig. 2), colonies are defined at the 48hr time point to avoid bias from significant colony merging after that point. The majority of the lineages maintain a Nanog::GFP signal.

File Name: Supplementary Movie 2
Description: Similar to Sup Movie 1, for cells differentiated for 0 hours, reseeded under Serum/LIF (0h – dox). A few of the colonies show mixed phenotype.

File Name: Supplementary Movie 3
Description: Similar to Sup Movie 1, for cells differentiated for 48 hours, and reseeded under Serum/LIF +OSKM (48h + dox). All four lineage types can be seen (GFP+, GFP mixed, GFP- ab+, GFP- ab-).

File Name: Supplementary Movie 4
Description: Similar to Sup Movie 1, for cells differentiated for 48 hours, reseeded under Serum/LIF (48h – dox). Only a few lineages acquire a GFP+ state.

File Name: Supplementary Movie 5
Description: Similar to Sup Movie 1, for cells differentiated for 96 hours, and reseeded under Serum/LIF +OSKM (96h + dox). At this point, none of the lineages reverts to a Nanog+ state, and no large colonies are formed.

File Name: Supplementary Movie 6
Description: Similar to Sup Movie 1, for cells differentiated for 96 hours, reseeded under Serum/LIF (96h – dox). Behavior is similar to the 96h + dox case.
